# Stress-Induced Mast Cell Activation in Glabrous and Hairy Skin

**DOI:** 10.1155/2014/105950

**Published:** 2014-05-08

**Authors:** Constantin Căruntu, Daniel Boda, Sorin Musat, Ana Căruntu, Eugen Mandache

**Affiliations:** ^1^Department of Physiology, “Carol Davila” University of Medicine and Pharmacy, 8 Eroii Sanitari Boulevard, 050474 Bucharest, Romania; ^2^Dermatology Research Laboratory, “Carol Davila” University of Medicine and Pharmacy, 22-24 Gr. Manolescu, 0111234 Bucharest, Romania; ^3^Themis Pathology SRL, 030447 Bucharest, Romania; ^4^“Dan Theodorescu” Oral and Maxillofacial Surgery Hospital, 17-21 Calea Plevnei, 010221 Bucharest, Romania; ^5^“Carol Davila” Clinical Hospital of Nephrology, Nephropathology Department, 4 Calea Grivitei, Sector 1, 010731 Bucharest, Romania

## Abstract

Mast cells play a key role in modulation of stress-induced cutaneous inflammation. In this study we investigate the impact of repeated exposure to stress on mast cell degranulation, in both hairy and glabrous skin. Adult male Wistar rats were randomly divided into four groups: Stress 1 day (*n* = 8), Stress 10 days (*n* = 7), Stress 21 days (*n* = 6), and Control (*n* = 8). Rats in the stress groups were subjected to 2 h/day restraint stress. Subsequently, glabrous and hairy skin samples from animals of all groups were collected to assess mast cell degranulation by histochemistry and transmission electron microscopy. The impact of stress on mast cell degranulation was different depending on the type of skin and duration of stress exposure. Short-term stress exposure induced an amplification of mast cell degranulation in hairy skin that was maintained after prolonged exposure to stress. In glabrous skin, even though acute stress exposure had a profound stimulating effect on mast cell degranulation, it diminished progressively with long-term exposure to stress. The results of our study reinforce the view that mast cells are active players in modulating skin responses to stress and contribute to further understanding of pathophysiological mechanisms involved in stress-induced initiation or exacerbation of cutaneous inflammatory processes.

## 1. Introduction

Inflammatory skin diseases such as psoriasis, atopic dermatitis, seborrheic eczema, acne, prurigo nodularis, lichen planus, chronic urticaria, rosacea, and alopecia areata are common disorders involving increased medical and socioeconomic costs. Moreover, skin inflammation is involved in skin cancer, wound healing, and other major cutaneous pathophysiological processes. Thus, the mechanisms and microscopical changes involved in cutaneous inflammatory processes are topics of major interest in scientific research [1, 2].

One of the main factors that can be involved in the onset or worsening of skin inflammation is psychological stress [3–7] and mechanisms by which stress can cause activation or modulation of skin inflammatory processes are still not completely understood. There are complex interconnections between nervous system and skin, including direct bidirectional communication pathways through peripheral cutaneous nervous system and indirect pathways through endocrine and immune system [8]. Numerous interactions were identified between nervous system and various immune cells such as mast cells, neutrophils, macrophages, and T cells [9, 10] but mast cells appear to be the key element in modulation of stress-induced cutaneous inflammatory processes. Thus, there is a close anatomical association between mast cells and cutaneous nerve fibers [11] and mast cells activation plays an important role in the process of neurogenic inflammation [12]. Cutaneous neurogenic inflammation is a complex process characterized by increased vascular permeability and vasodilation [13–15] induced by neuromediators such as substance P (SP) and calcitonin gene-related peptide (CGRP) that are released from cutaneous nerve endings. These neuromediators can also induce mast cell degranulation with subsequent histamine and proinflammatory cytokines release that can amplify the inflammatory skin process [16]. Previous studies have shown that stress exposure induces a significant increase in dermal mast cells degranulation, in number/density of nerve fibers immunoreactive for SP and contacts between mast cells and these fibers [17, 18]. Stress-induced increase in mast cell degranulation occurs rapidly [19] and is maintained over 7 days after exposure to stress [20]. Stress-induced mast cell degranulation is dependent on corticotropin-releasing hormone (CRH) but probably also involves the action of SP and neurotensin. Stress does not trigger an explosive mast cell degranulation, specific to anaphylactic reactions, but appears to act in a more subtle manner on mast cells, inducing predominantly intragranular changes [12].

In most previous studies stress exposure was limited to a single exposure and effects on dermal mast cells were investigated immediately after exposure to stress [12, 19] at 2 days [17, 18, 21] or 7 days after stress exposure [20]. Also, previous studies that investigated the effects of stress on cutaneous mast cells were restricted to hairy skin.

In this study we aim to investigate the impact of repeated exposure to stress on mast cell degranulation in both glabrous and hairy skin, thus allowing a dynamic study of stress effects on these important players in cutaneous inflammatory processes.

## 2. Material and Methods

### 2.1. Experimental Protocol

The study was conducted on 29 adult male Wistar rats, weighing 250–350 g, that were housed in clean cages, with a maximum of 5 rats per cage, in standard experimental conditions, including light-dark cycle of 12 h/12 h, temperature 22 ± 2°C, and free access to food and water. Experiments were conducted in accordance with European Guidelines on Laboratory Animal Care and were approved by the local ethics committee.

Rats were randomly divided into four groups: Stress 1 day (*n* = 8), Stress 10 days (*n* = 7), Stress 21 days (*n* = 6), and Control (*n* = 8). We used a restraint stress protocol in which the rats were placed in a well-ventilated restrainer that prevented movement without harming the animal. Rats from Stress 1 day group had a single exposure to restraint stress with duration of 2 hours. Rats from Stress 10 days and 21 days groups were exposed to stress protocol for 2 hours per day for a total of 10 and, respectively, 21 consecutive days. Rats from the Control group were maintained on standard experimental conditions.

After 24 h from the last exposure to stress tissue fragments of both hairy and glabrous skin from all animals were collected in order to assess cutaneous mast cell degranulation by light microscopy. Hairy skin was harvested from the ears and glabrous skin was harvested from the plantar region of posterior limbs. Additional samples of hairy and glabrous skin of approximately 1 mm^3^ were collected for ultrastructural examination via transmission electron microscopy (TEM). All reagents used for the experiments were purchased from Sigma-Aldrich (St. Louis, MO, USA), unless otherwise specified.

### 2.2. Preparation of Tissue Samples for Optical Microscopy

Fixation of tissue samples was done with 4% formaldehyde solution in phosphate buffered saline (PBS) 150 mM, pH 7.5, for a duration of 24 ± 1 hours. After dehydration through graded ethanols and clearing in butanol, representative fragments of glabrous and hairy skin were embedded in paraffin in a sectionable vegetal matrix, numbering a maximum of 12 samples per block, according to “tissue microarray” technology, as previously described [22]. The resulting paraffin blocks were sectioned semiserially at 4-5 *μ*m.

### 2.3. Histochemical Stains to Highlight Mast Cells

Three different types of staining techniques were used to identify mast cells.

1. *Giemsa Staining*. Briefly, after dewaxing and rehydration, sections were stained for 60 minutes in Giemsa solution (Bio-Optica, Milan, Italy) in PBS (pH 6.8). Sections were differentiated with 1 : 1000 glacial acetic acid, dehydrated, cleared, and mounted.
2. *Acidified Toluidine Blue Staining* [23]. Deparaffinized and rehydrated sections were stained for 5 minutes in 0.02% toluidine blue solution, pH 3.2.
3. *Alcian Blue-Safranin O Staining* [24]. Following dewaxing and rehydration, sections were stained for 30 minutes with Alcian blue-Safranin O (0.36% and 0.18%) in 0.1 M acetate buffer solution, pH 1.42.

### 2.4. Histomorphometric Analysis of Optical Microscopy Images

The resulting slides were examined and photographed in a blinded fashion on an Olympus CKX41 microscope equipped with an SLR Olympus E330 digital camera. At least 5 random high power fields (400x) for every test sample were selected and photographed. The images were acquired in a format of 3136 × 2352 pixels using QuickPhoto 2.3 software and stored in the TIFF format. We performed the histomorphometric analysis of images in a blinded manner, using the image analysis software ImageJ 1.45. In the dermis we evaluated the percentage of degranulated mast cells from the total number of mast cells, in both glabrous and hairy skin. Degranulated mast cells were defined as mast cells with a reduced staining intensity in the cytoplasm and presenting extracellular granules in close proximity [12, 25–27]. A minimum of 5 microscopic fields and a minimum of 10 cells were assessed for each rat and each type of tissue.

### 2.5. Ultrastructural Cutaneous Aspects Assessed with Electron Microscopy

Fixation of tissue fragments was performed with 4% buffered glutaraldehyde followed by 1% buffered osmic acid. The 1 mm^3^ fragments were dehydrated in graded alcohols and embedded in Epon. Thin sections of 80 nm, double stained with uranyl acetate and lead citrate, were prepared on a LKB ultramicrotome. The study was performed with a JEOL JEM 1011 electron microscope at 80 kV. All skin compartments were examined, with particular focus on mast cells, skin blood vessels, and nerve fibers.

### 2.6. Statistical Analysis

The normality and homogeneity distribution of data were assessed with Bartlett's test. We used Kruskal-Wallis test followed by 2-tailed multiple comparison test to evaluate differences between experimental groups. For each group, the results were presented as mean ± standard deviation (SD). *P* values <0.05 were considered significant.

## 3. Results

**Figure 1:**
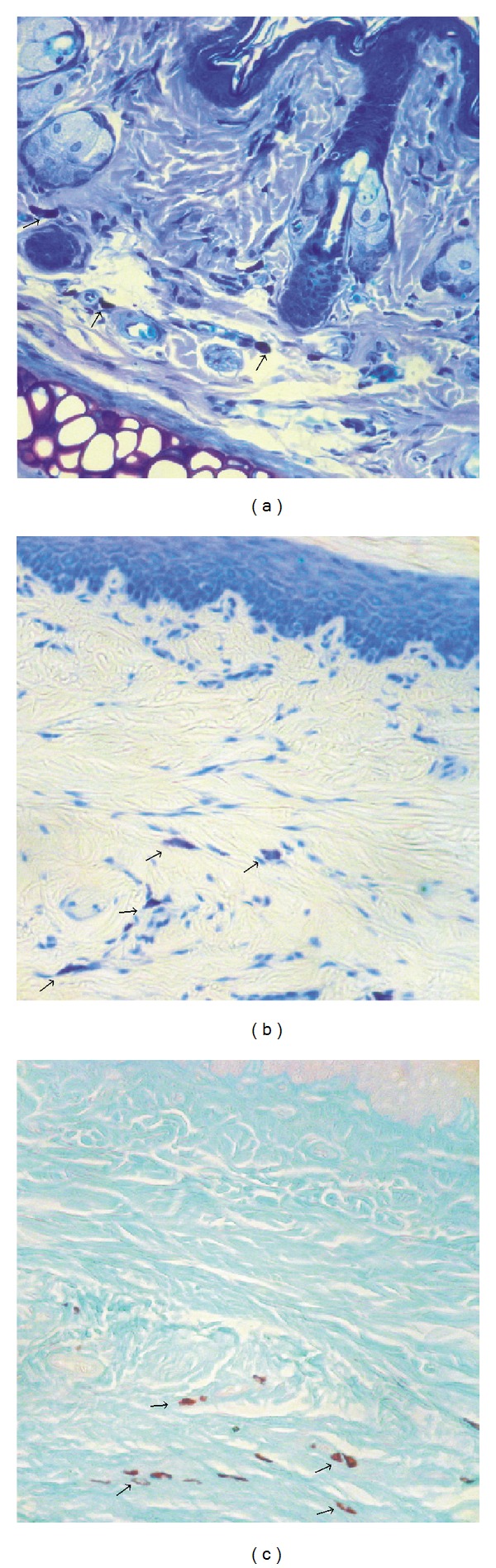
Histochemical aspect of mast cells (→) with (a) Giemsa staining; (b) acidified toluidine blue staining; (c) Alcian blue-Safranin O staining. Magnification 400x.

We carried out a preliminary assessment of the tissue slides processed using the three methods mentioned above for histochemical identification of mast cells: Alcian blue-Safranin O staining, acidified toluidine blue staining, and Giemsa staining (Figure 1). The most conclusive data were obtained on histological preparations stained with Alcian blue-Safranin O, and this method was further used to evaluate mast cell degranulation in skin tissue.

**Figure 2:**
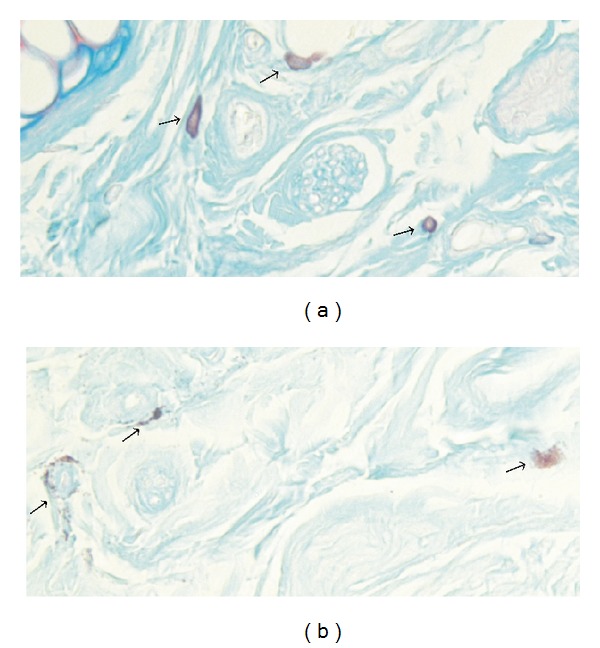
(a) Normal mast cells (→) localized near vasculonervous structures in hairy skin. Alcian blue-Safranin O staining. 400x magnification; (b) Degranulated mast cells (→) in immediate vicinity of vasculonervous structures in glabrous skin. Alcian blue-Safranin O staining. Magnification 400x.

In all examined tissue samples mast cells were present in the dermis of both glabrous and hairy skin. Mast cells were typically located at the dermoepidermal junction and dermis-hypodermis interface. In addition, we identified an important number of mast cells organised near cutaneous nerve structures or blood vessels (Figures 2(a) and 2(b)).

**Figure 3:**
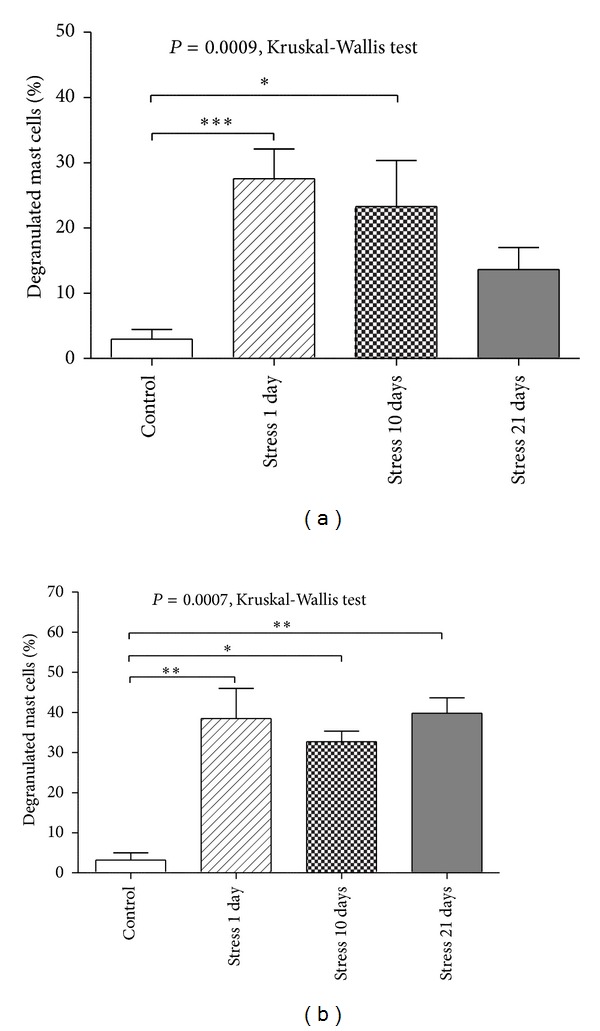
Comparative analysis of the proportion of degranulated mast cells. (a) In glabrous skin, brief exposure to stress induced a significant increase in the proportion of degranulated mast cells and prolonged exposure to stress was associated with a gradual return to normal value of degranulated mast cells. (b) In hairy skin, acute exposure to stress induced an amplification of mast cell degranulation process that was maintained after prolonged exposure to stress. Error bars represent the standard error of the mean (SEM). *P < 0.05, **P < 0.01, ***P < 0.001, two-tailed multiple comparison test.

In glabrous skin, stress exposure increased the proportion of degranulated mast cells (*P* = 0.0009, Kruskal-Wallis test). Initial growth was important, from 2.95 ± 4.31% in the Control group to 27.57 ± 12.80% in the Stress 1 day group (*P* = 0.00081, two-tailed multiple comparison test). Prolonged exposure to stress was associated with a gradual return to normal values of the proportion of degranulated mast cells. Thus, for rats from Stress 10 days group the percentage of degranulated mast cells was 23.31 ± 18.65%, a value that is still significantly higher than Control group (*P* = 0.01649, two-tailed multiple comparison test). However, in laboratory animals from Stress 21 days group the percentage of degranulated mast cells was only 13.65 ± 8.16%, a value that is not statistically significant compared to Control group (*P* = 0.23840, two-tailed multiple comparison test) (see Figure 3(a)).

In hairy skin, stress exposure also induced an increase in mast cells degranulation (*P* = 0.0007, Kruskal-Wallis test). As with glabrous skin, a single stress exposure led to a significant increase in mast cell degranulation, from 3.20 ± 5.04% in the Control group to 38.46 ± 21.23% in the Stress 1 day group (*P* = 0.00205, two-tailed multiple comparisons test). However, in contrast to glabrous skin, after prolonged stress exposure the percentage of degranulated mast cells in hairy skin remained high when compared to Control group. Thus, in Stress 10 days group the proportion of degranulated mast cells was 32.70 ± 7.01% (*P* = 0.02989, two-tailed multiple comparison test) and in Stress 21 days group the percentage of degranulated mast cells was 39.76 ± 9.42% (*P* = 0.00496, two-tailed multiple comparison test) (see Figure 3(b)).

**Figure 4:**
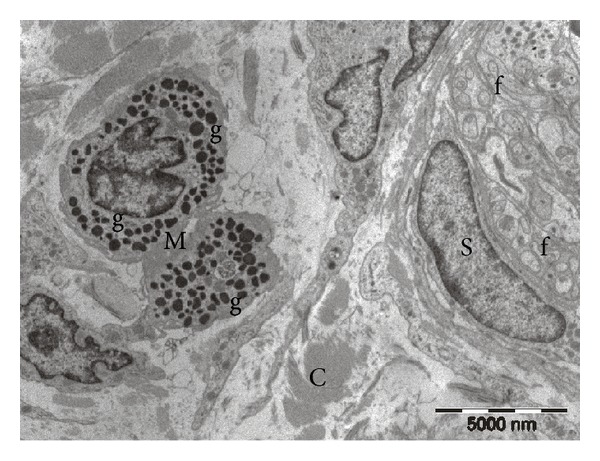
Electron microscopy image from the rat control group. Two dermal mast cells (M) with uniform electron-dense granules (g), placed near a nerve bundle containing a Schwann cell (S) and unmyelinated nerve fibers (f). Collagen (C) in the surrounding background.

Investigation of glabrous and hairy skin samples by transmission electron microscopy confirmed the presence of mast cells in the dermis, adjacent to nerve fascicles and blood vessels. Often the distance between mast cells and nervous or vascular structures was less than 1–3 *μ*m. In the samples from Control group, both in hairy and glabrous skin, mast cells had a normal appearance, with numerous intracytoplasmic electron-dense granules, uniform appearance, round-oval shape, and multiple cytoplasmic extensions (see Figure 4).

**Figure 5:**
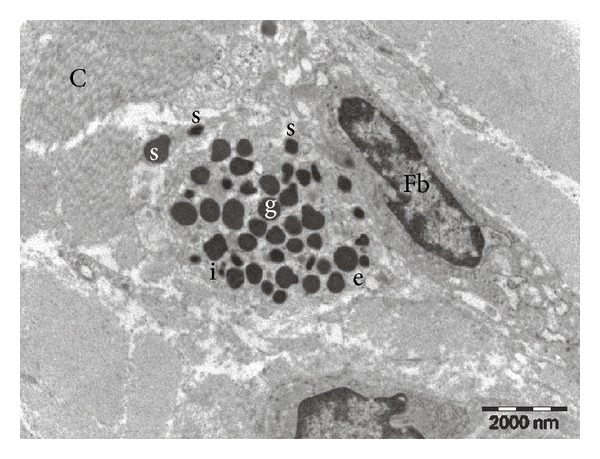
Electron microscopy picture from Stress 1 day rat group. Mast cell with dense granules (g), granules emptied of content (e), granules showing altered electron densities (i), and granules on the verge of releasing the content into the extracellular space (s). Collagen (C) and fibroblast (Fb).

In rats subjected to a single exposure to stress, the electron microscopic appearance of dermal mast cells from both types of skin was changed compared to normal (see Figure 5). Elements specific for mast cell degranulation were observed, such as (i) granules that appear to be released in the extracellular environment, (ii) granules that are about to release their contents into extracellular environment, and (iii) merged intracytoplasmic granules. In addition, mast cells with irregular granules or granules emptied of content were identified, indicating a partial mast cell degranulation process.

**Figure 6:**
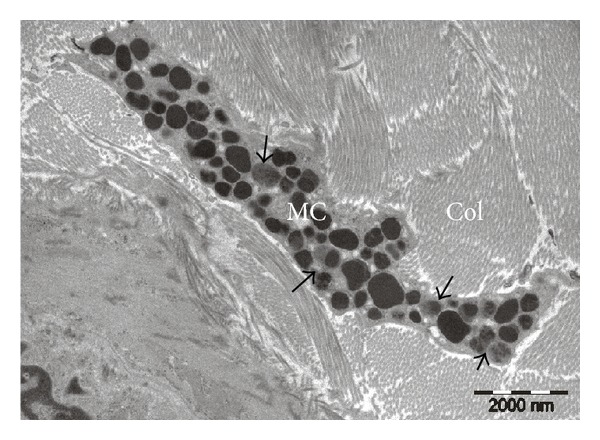
Electron micrograph from Stress 21 days rat group. A dermal mast cell (MC) with dense granules and granules that show altered electron densities (arrows), indicating a process of mast cell activation. Collagen fibers (Col) in the surrounding background.

Electron microscopic evaluation of glabrous and hairy skin harvested from rats from Stress 10 days group showed predominantly mast cells with intracytoplasmic granules with low or uneven electron density, suggestive of either activation or partial degranulation. Also, sporadic granules that seemed on the verge of eliminating their content in the extracellular environment were identified. A similar appearance of mast cells was observed in glabrous and hairy skin samples collected from rats belonging to the Stress 21 days group (see Figure 6).

## 4. Discussion

Psychological stress may be involved in the modulation of cutaneous inflammation and its effects may vary depending on the duration and characteristics of the stressor. Thus, chronic stress has an immunosuppressive effect, while acute stress, although it induces a significant decrease in circulating immune cells by redirecting them to the skin, stimulates cell-mediated immunity in cutaneous tissue [28]. In addition, exposure to stress can lead to modulation of skin peptidergic innervation [21] and can induce the release of neuropeptides from peripheral nerve endings, leading to activation of neurogenic inflammation [17, 20], a process that involves mast cells activation [12]. Inflammatory processes of various skin disorders triggered or exacerbated by stress, such as atopic dermatitis and psoriasis, also have mast cell activation as a key mechanism [29–33].

The important role of mast cells in stress-induced activation of skin inflammation is suggested by their strategic location near cutaneous nerve structures and blood vessels and their complex interconnections with nerve fibers, immune cells, and keratinocytes [11, 34, 35].

Despite this, there are only a limited number of studies that investigated the effect of chronic stress on skin mast cells. One experimental study in rats showed that there is no statistically significant difference between control and chronic stressed groups regarding paw oedema induced by a mast cell degranulator compound [36]. Another study in patients with psoriasis showed that in noninvolved hairy skin of the arms and on the back chronic stress is associated with an increased number of inflammatory cells expressing neurokinin 1 receptor, most of them being mast cells [37].

The effect of acute stress on mast cell degranulation has been investigated in murine hairy skin. Previous studies, limited to a single stress exposure, highlighted a significant increase in dermal mast cells degranulation shortly after exposure to stress [12, 19] and lasting for over one week [20].

Our research assessed the impact of repeated stress exposure on mast cell degranulation in hairy and glabrous skin, moving a step forward from previous studies. We used a restraint stress model commonly used in scientific research, which induces a moderate intensity stress, largely psychological [38, 39]. Restraint stress has been used to study the effects of both acute [28, 40] and chronic stress [40, 41]. To study the acute stress, animals may be exposed to restraint stress in a single experimental session with duration between 60 minutes [40] and 2 hours [28]. For chronic stress induction, the duration of restraint may also vary from repeated exposure of 60 minutes per day for 10 consecutive days [40] to prolonged exposure, of 6 hours per day for 21 consecutive days [41]. Thus, our experimental group with a single experimental session of 2 hours may be classified as acute stress while the results achieved after a total of 10 and, respectively, 21 consecutive days of stress exposure are investigating the effects of chronic stress.

In our study acute stress induced a significant increase in the proportion of degranulated mast cells in both hairy and glabrous skin, confirming the results of previous research.

However, the effects of chronic stress were different in the two types of skin investigated. In glabrous skin chronic stress was associated with a slow return towards normal values of mast cells degranulation. In contrast, in hairy skin, the percentage of degranulated mast cells remained high even after 21 days of stress exposure. Another feature revealed in the chronic stress groups was the phenomenon of partial degranulation (mast cell activation), as described in previous studies [42, 43], in which the content of granules is only partially released in the extracellular environment.

The distinct impact of chronic stress on mast cells from glabrous and hairy skin may be explained by the different pattern of innervation [44–46] regarding both the myelinated nerve fibers [47] and the unmyelinated nociceptive nerve fibers [48]. Moreover, different functional features of A*δ* and C nerve fibers innervating the human glabrous and hairy skin have been previously reported [49]. Unmyelinated type C and thin myelinated type A*δ* nerve fibers are rich in various neuropeptides, such as SP and CGRP, and, together with mast cells, are key players in cutaneous neurogenic inflammation [50–52].

As already mentioned, there is a close connection between cutaneous nerve fibers and mast cells from both the morphological and the functional perspective. Thus, mast cells can be activated by neuropeptides, such as SP [53–55], neurotensin [12, 56], nerve growth factor (NGF) [57, 58], and pituitary adenylate cyclase-activating polypeptide (PACAP) [59] released from skin nerve endings.

The effect of stress on skin mast cells appears to be mediated by the release of neuropeptides from cutaneous nerve endings. Stress induces a release of NGF and neurotensin [60, 61] and increases SP-positive nerve fibers and their contacts with mast cells in skin [17, 18, 21].

Another key player in stress-induced mast cell activation is CRH [12]. Under stress, CRH is secreted from hypothalamus and can also be released in the skin by activated nerve endings and local immune cells [62–65]. CRH activates skin mast cells in murine [66] and human skin [67] and also induces the development of mast cells from precursors in the human hair follicle mesenchyme [68].

The complexity of the relationships between nerve fibers and mast cells is further evidenced by research showing that mast cells may contain SP [69], NGF [70], and CRH [71]. Moreover, substances released by mast cells, such as histamine, serotonin, and proinflammatory cytokines, may have a modulatory action on nerve fibers function and neuropeptides effects [50, 72, 73].

## 5. Conclusions

The impact of stress on mast cell degranulation seems to be different, depending on the type of skin, glabrous or hairy, as well as on the duration of stress exposure. In the hairy skin acute stress induces an increased mast cell degranulation that persists even after prolonged exposure to stress, while in glabrous skin a short-term stress exposure has a strong stimulating effect of mast cell degranulation that subsides in intensity as exposure to stress persists. Thus, our findings could provide an explanation for the varying impact of stress on different dermatological diseases depending on the location of skin lesions or on the duration of exposure to stress [74, 75].

Our study contributes to the further understanding of the mechanisms involved in the initiation or exacerbation of cutaneous pathophysiological processes by stress. This is an area of active interest for the international scientific community that currently invests many efforts in developing more active and more specific pharmacological agents targeting the association of stress with inflammatory dermatological disorders.
